# Maternal Obesity during the Preconception and Early Life Periods Alters Pancreatic Development in Early and Adult Life in Male Mouse Offspring

**DOI:** 10.1371/journal.pone.0055711

**Published:** 2013-01-31

**Authors:** Isabele Bringhenti, Jessica Andrade Moraes-Teixeira, Michelle Rabello Cunha, Fernanda Ornellas, Carlos Alberto Mandarim-de-Lacerda, Marcia Barbosa Aguila

**Affiliations:** Laboratory of Morphometry, Metabolism and Cardiovascular Disease, Biomedical Centre, Institute of Biology, State University of Rio de Janeiro, Rio de Janeiro, Rio de Janeiro, Brazil; National Cancer Institute, United States of America

## Abstract

Maternal obesity induced by a high fat (HF) diet may program susceptibility in offspring, altering pancreatic development and causing later development of chronic degenerative diseases, such as obesity and diabetes. Female mice were fed standard chow (SC) or an HF diet for 8 weeks prior to mating and during the gestational and lactational periods. The male offspring were assessed at birth, at 10 days, and at 3 months of age. The body mass (BM) gain was 50% greater before pregnancy and 80% greater during pregnancy in HF dams than SC dams. Dams fed an HF diet showed higher oral glucose tolerance test (OGTT), blood pressure, serum corticosterone, and insulin levels than dams fed SC. At 10 days of age and at 3 mo old the HF offspring showed greater BM and higher blood glucose levels than the SC offspring. The mean diameter of the islets had increased by 37% in the SC offspring and by 155% in the HF offspring at 10 days of age. The islet mass ratio (IM/PM) was 88% greater in the HF offspring at 10 days of age, and 107% greater at 3 mo of age, compared to the values obtained at birth. The HF offspring had a beta cell mass (BCM)/PM ratio 54% lower than SC offspring at birth. However, HF offspring displayed a 146% increase in the BCM/PM ratio at 10 days of age, and 112% increase at 3 months of age than values at birth. A 3 mo of age, the HF offspring showed a greater OGTT and higher levels of than SC offspring. In conclusion, a maternal HF diet consumed during the preconceptional period and throughout the gestational and lactational periods in mice results in dramatic alterations in the pancreata of the offspring.

## Introduction

The global rise in the prevalence of overweight and obese patients is paralleled by an alarming increase in the incidence of overweight pregnant women [1]. The consumption of energy-dense food is recognized as a contributing factor to the etiology of the present obesity epidemic and is partially responsible for the increase in women being overweight during pregnancy [2].

Maternal obesity at conception and during the gestational period has been proposed to lead to developmental programming of excess weight gain and adiposity in offspring [3], [4]. Furthermore, the consequences of maternal obesity and excess maternal nutrition during gestation and lactation for adult progeny include metabolic disorders involving abnormal glucose homeostasis, reduced whole-body insulin sensitivity, impaired beta cell insulin secretion and changes in the structure of the pancreas [5], [6]. Metabolic disruption is strongly associated with deleterious effects on beta cell development and function [7]. Moreover, hyperglycemia and hyperinsulinemia are observed in the offspring of mothers exposed to a high fat (HF) diet and are associated with reduced insulin secretion by beta cells and whole-body insulin resistance by adulthood [6], although this conclusion is not unanimously accepted [8]. One explanation for the different effects associated with maternal HF diets in the offspring is that different HF diets have been produced with fat contents between 20–60% containing animal-derived fats, such as lard or beef tallow, or plant oils, such as canola or olive oil [9], [10]. Such HF diets have been tested in different animal models with various fat administration protocols, leading to a better understanding the effect of providing HF diets to mothers and the consequences in the progeny [11], [12].

Studies on maternal obesity in rodents have suggested that diet-induced maternal obesity can predispose offspring to impaired pancreatic function [13], [14], possibly involving the transcription factor necessary for pancreatic development and beta cell maturation, or Pdx1, also known as insulin promoter factor 1 (in rodents) and as Ipf1 (in humans), representing the gene encoding it [15]. However, the variability of the HF diets and animal models used in studies on this topic makes it difficult to compare their findings. Therefore, the impact of diet-induced maternal obesity in different stages of development (such as immediately before and during pregnancy or during lactation) on the development of the pancreas in offspring is still a matter of debate.

Thus, we hypothesize that an HF diet administered to mothers beginning in the preconceptional period and reinforced in the gestational and lactational periods could alter the structure and pathophysiology in the pancreas during its development. These consequences will appear soon after birth, continuing and being aggravated in adulthood.

## Materials and Methods

### Animals and maternal diet

This study was carried out in strict accordance with the recommendations of the Guide for the Care and Use of Laboratory Animals of the National Institutes of Health. The protocol was approved by the Committee on the Ethics of Animal Experiments of the State University of Rio de Janeiro (Permit Number: CEUA 053/10), and all efforts were made to minimize suffering.

Four-week-old female C57BL/6 mice were randomly assigned into standard chow (SC) or high fat (HF) diet groups (n = 20 per group). The animals were housed in temperature- and humidity-controlled cages with a constant 12/12-hour light-dark cycle. The SC group was fed a standard chow diet with 64% of the calories coming from carbohydrates, 19% of the calories from protein, and 17% of the calories from lipids (70 g soybean oil/kg food). The HF diet group was fed an HF diet in which 32% of the calories were derived from carbohydrates, 19% of the calories from protein, 32% of the calories from animal lard (200 g/kg food) and 17% of the calories from other lipids (70 g soybean oil/kg food). The vitamin and mineral contents of both diets were identical and followed the standards for rodent diets recommended by the American Institute of Nutrition to support growth during the pregnancy, lactation and post-weaning periods (AIN-93G) [16]. Both the SC and HF experimental diets were manufactured by PragSolucoes (Jau, SP, Brazil).

**Figure 1:**
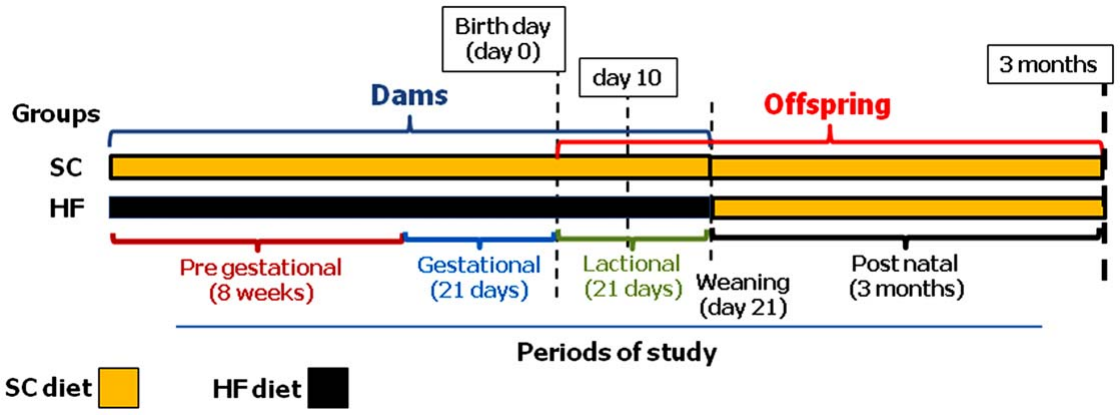
Timeline of the study. Female mice received standard chow (SC) or a high fat (HF) diet for 8 weeks prior to and during the gestational and lactational periods. The male offspring were assessed at birth and at 10 days and 3 months of age.

Figure 1 shows the timeline of the experiment. After 8 weeks on the two diets, 12-week-old female mice were mated with male C57BL/6 mice that were fed an SC diet. Day 0 of gestation was determined by observing the formation of a vaginal plug, and the pregnant dams were maintained on their respective diets throughout pregnancy and lactation. Maternal body mass (BM) was measured weekly, and dietary intake was measured daily over the course of the experiment. Fresh chow was provided daily, and any remaining chow from the previous day was discarded. Food consumption was determined as the difference between the food supplied and the amount of food remaining in the cage. The energy intake was estimated as the product of food consumption and the energy content of the diet.

After the birth of the offspring, the litter size in each cage was randomly adjusted to seven pups (four males, when possible) to ensure adequate and standardized nutrition until the pups were weaned [17]. Offspring sex was assessed by measuring the anogenital distance [18].

**Figure 2:**
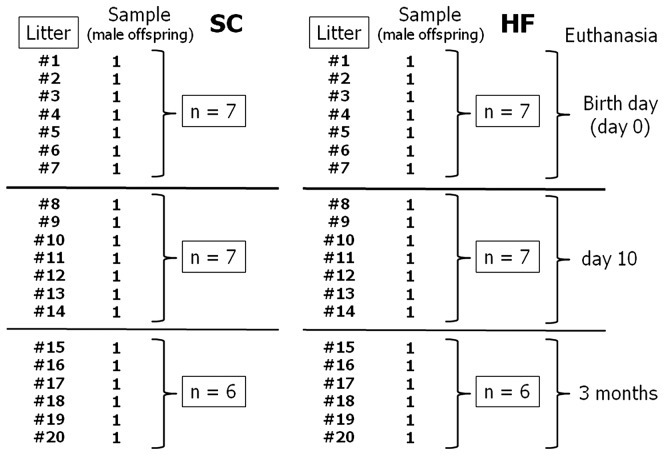
Sampling design for the study.

The offspring were labeled according to the maternal diet: SC pups were birthed from mothers fed SC, whereas HF pups were birthed from mothers fed the HF diet. One male from each litter was randomly assigned to the experimental groups. In summary, seven male offspring coming from seven different SC litters were killed at birth day (n = 7). The other offspring in these SC litters were killed at 10 days of age (n = 7). The remaining animals were allowed to develop until reaching three month of age, when they were also killed (n = 6). The same protocol was applied among seven different HF litters (Fig. 2). Thus, the SC and HF pups assigned to the experimental groups were analyzed at three different ages: day 0 (birth day), day 10 after birth, when organogenesis should be complete in rodents [19], and at adult age (at three months of age). The BM and naso-anal length were evaluated at birth, at postnatal day 10 and at three months of age. From weaning to 3 months of age, the animals were fed an SC diet until euthanasia.

### Mothers

#### Blood pressure

The systolic blood pressure (BP) was measured every Thursday at 13:00 h prior to pregnancy in conscious animals using the noninvasive method of tail-cuff plethysmography (Letica LE 5100, Harvard/Panlab, Barcelona, Spain).

#### Oral glucose tolerance test, serum corticosterone, and insulin determination

The oral glucose tolerance test (OGTT) was performed after 6 hours of fasting in dams two days before mating. To induce an overload of glucose, a known amount of hypertonic glucose (1 g/kg body weight) was administered via orogastric gavage. Blood was collected from the tail at 0, 15, 30, 60 and 120 min following glucose administration, and blood glucose was measured using a glucometer (Accu-Chek, Roche, Germany). The area under the curve (AUC, arbitrary units, a.u.) was measured to assess glucose tolerance. The blood glucose level at time 0 was considered the fasting glucose level.

After weaning the pups, the dams were anesthetized via intraperitoneal injection of sodium pentobarbital (150 mg/kg). Then, an incision was made in the chest wall exposing the heart, and blood samples were rapidly obtained via cardiac puncture of the right atrium.

The inguinal fat pad located between the lower part of the rib cage and the mid-thigh was considered subcutaneous fat. The retroperitoneal fat and ovarian fat were considered the intra-abdominal fat pad. Both the subcutaneous and intra-abdominal fat pads were carefully dissected and weighed. The adiposity index was determined as the ratio between the sum of the intra-abdominal and subcutaneous masses divided by the total BM [20].

Serum was obtained from the dams for subsequent corticosterone and insulin analyses. The serum corticosterone and insulin concentrations were measured using a commercially available enzyme-linked immunosorbent assay kit (corticosterone ELISA kit ab 108821; Abcam Inc., Cambridge, USA, Rat/Mouse Insulin ELISA kit Cat. #EZRMI-13K, Millipore, Missouri, USA). All samples were analyzed in duplicate, and the intraassay coefficient of variation was 4.4% for corticosterone detection and 5.1% for insulin detection.

### Offspring

At birth and at postnatal day 10, male offspring were killed by decapitation, and their blood glucose was measured using a glucometer (Accu-Chek, Roche, Germany). The OGTT was performed in the male offspring group at three months of age (as described for the mothers). The animals were then deeply anesthetized (sodium pentobarbital, 150 mg/kg i.p.) and killed via exsanguination. Their plasma was obtained for subsequent biochemical analysis. The plasma concentrations of insulin were evaluated using the Milliplex mouse serum adipokine panel kit (MADPK-07-71K) with Luminex xMAP equipment (Millipore, Billerica, MA, USA).

The pancreata were excised in all experimental groups, then weighed and immersed in freshly prepared fixative (1.27 mol/L formaldehyde in 0.1 M phosphate buffer, pH 7.2) for 48 hours at room temperature.

### Pancreas

#### Immunohistochemistry

For immunohistochemical analysis, pancreas sections from all age groups were incubated with guinea pig anti-insulin (A0564, Dako, Glostrup, Denmark) at a dilution of 1∶100. At day 0 and 10 days of age, pancreas sections were incubated with a monoclonal mouse anti-proliferating cell nuclear antigen (PCNA) (Clone PC10, Dako) at a dilution of 1∶100 and with polyclonal rabbit anti-Pdx1 (Pancreatic and duodenal homeobox 1, AB 3503) at a dilution of 1∶100, followed by incubation with a biotin–streptavidin complex (K0679; Universal DakoCytomation LSAB + Kit, Peroxidase, Glostrup, Denmark). Pancreas sections were developed with 3, 30-diaminobenzidine tetrachloride (K3466, DAB, DakoCytomation), and the sections were counterstained with Mayer's hematoxylin.

Digital images of the stained slices were obtained using a Lumenera Infinity 1–5 digital camera (Lumenera Co., Ottawa, ON, Canada) mounted on a Leica DMRBE microscope (Wetzlar, Germany) (TIFF format, 1280×1024 pixels).

#### Immunofluorescence

Antigen retrieval was accomplished using citrate buffer, pH 6.0, at 60°C for 20 min and blocked with ammonium chloride, 2% glycine, and phosphate-buffered saline, pH 7.4 (PBS). Pancreatic sections were incubated with guinea pig anti-insulin (A0564, Dako). Primary antibodies were diluted 1∶50 in blocking buffer (PBS/1% bovine serum albumin, BSA), and incubations were conducted overnight at 4°C. The samples were then incubated for 1 h at room temperature with a fluorochrome-conjugated secondary antibody, goat anti-guinea pig IgG-Alexa 546 (Invitrogen, Molecular Probes, Carlsbad, CA, USA), diluted 1∶50 in PBS/1% BSA. After rinsing in PBS, the slides were mounted with DAPI Nucleic Acid Stain and SlowFade Antifade (Invitrogen, Molecular Probes, Carlsbad, CA, USA). Indirect immunofluorescence images were captured using confocal microscopy (Zeiss Confocal Laser Scanning Microscopy – LSM 510 Meta, Germany). These images were used to measure the inter-cell nuclear distance as a measure of the beta cell size to characterize cell hypertrophy [21].

#### Islet morphometry (at birth and at postnatal day 10, and at 3 mo of age)

Five-micrometer-thick sections were obtained from each pancreas at birth and at postnatal day 10 and stained with hematoxylin and eosin. Morphometric analyses were performed on digital images acquired with the above-mentioned equipment using Image Pro Plus software, version 7.01 (Media Cybernetics, Silver Spring, MD, USA). The largest and smallest diameters of the islets were measured to estimate the average islet diameter from at least 100 islets per group.

#### Islet volume density and islet mass (at birth, at postnatal day 10, and at 3 mo of age)

The islet volume density (Vv[islet]) was estimated using a point counting system based on the ratio of points landing on an islet (Pp) and the total number of test points in the system, which was comprised of 49 test-points (P_T_) constructed with the on-line STEPAnizer system (www.stepanizer.com) [22]: Vv[islet] = Pp[islet]/P_T_ (100%). The islet mass (M[islet]) was obtained by multiplying Vv[islet] by the pancreatic mass [23].

#### Beta cell volume density and beta cell mass (at birth, at postnatal day 10, and at 3 mo of age)

The beta cell volume density (Vv[beta cell]) was estimated via image analysis using the density threshold selection tool applied to the insulin-positive areas of the islets expressed as a percentage of the islet (Image-Pro Plus version 7.01, Media Cybernetics, Silver Spring, MD, USA) [21]. Thus, beta cell mass (M[beta cell]) was estimated as the product of Vv[beta cell] and M[islet] [24], [25].

#### Islet PCNA density per area (at birth and at postnatal day 10)

The density per area of the PCNA was estimated considering a test area of 0.0289 mm^2^ constructed with the on-line STEPAnizer system [22]. All PCNA nuclei inside the frame that did not hit two consecutives lines of the frame considered “forbidden lines” were counted [26]. A sample of five slices containing two sections in each slice was counted in each group.

### Data Analysis

The data are expressed as the mean and the associated standard error of the mean. Differences between the groups were analyzed using one of the following tests: paired or unpaired Student's *t*-test, one-way ANOVA followed by the Holm-Sydak post-hoc test, or two-way ANOVA to determine the effects of diet and age. Fisher's exact test was used to test the frequencies of dams mated and mortality rates in the offspring. A *P*-value≤0.05 was considered statistically significant (GraphPad Prism version 6.01 for Windows, GraphPad Software, La Jolla California USA).

## Results

### Mothers

The success of the pregnancies (frequency of positive pregnancies) did not differ between the SC and HF groups of mothers (*P* = 1.00, odds ratio = 1.588, 95% confidence interval = 0.2355 to 10.71, Fisher's exact test).

#### Body mass, feed efficiency and adiposity index

**Figure 3:**
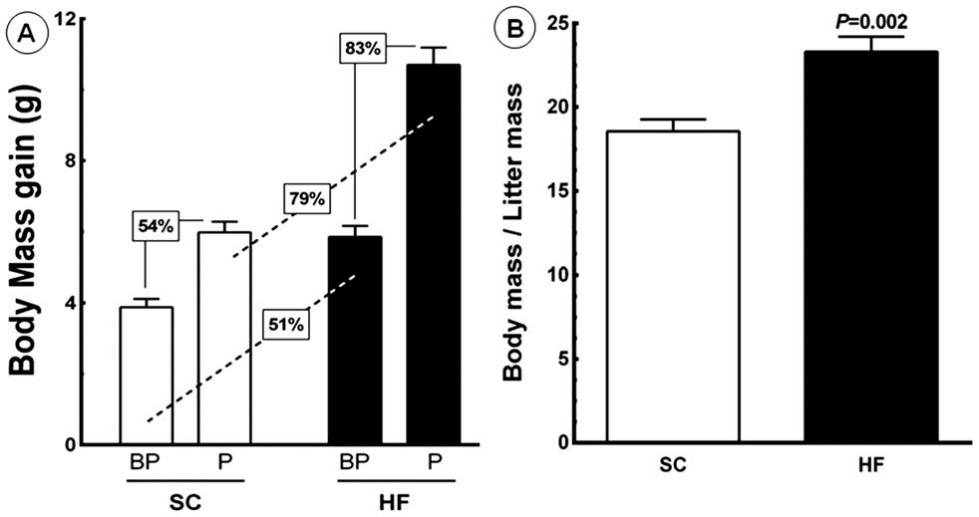
Maternal data. (A) body mass gain before pregnancy (BP) and during pregnancy (P). Significant differences between the groups are indicated with the percentage of increase (n = 10 animals per group; a paired *t*-test was used to compare the periods BP and P; one-way ANOVA was used to analyze all groups). (B) The body mass/litter mass ratio was different between the groups. Abbreviations: SC, standard chow group; HF, high fat diet group.

Figure 3A shows the BM gain in the dams over the course of the experiment. During the eight-week period before pregnancy, the BM gain for SC dams was 3.88±0.24 g, whereas the BM gain in HF dams was 5.85±0.31 g. During pregnancy, the BM gain for SC dams was 5.98±0.30 g, whereas the BM gain for HF dams was 10.70±0.49 g. Diet accounted for 37.95% of the differences observed and was a significant factor for weight gain (*P<*0.0001, two-way ANOVA). Pregnancy accounted for 40.96% of the differences observed and was a significant factor for weight gain (*P<*0.0001, two-way ANOVA). Moreover, there was an interaction between diet and pregnancy, which accounted for 6.41% of the observed differences and was significant (*P = *0.0003, two-way ANOVA). The maternal body mass was normalized for the litter mass (Fig. 3B). HF dams showed a higher maternal body mass to litter mass ratio than SC dams (+25.60%, 23.30±0.9 g *vs.* 18.55±0.7 g, *P*<0.05). However, the number of pups and pup mass were not different between SC and HF dams. The feeding efficiency (FE) was analyzed before and during pregnancy. Before pregnancy, the FE was 51% greater in HF dams (1.5±0.13 g/kJ) than in SC dams (1.0±0.06 g/kJ) (*P<*0.0039). During pregnancy, the FE was 256% higher in HF dams (7.12±0.48 g/kJ) than in SC dams (2.00±0.33 g/kJ) (*P<*0.0001). Notably, increases in FE paralleled BM gains during the same period (Tables 1 and 2).

**Table 1:** Data from dams fed standard chow (SC) or high fat (HF) diet expressed as mean and SEM.

Data	SC	HF	*P*-value
Feed efficiency before pregnancy, g/kJ	1.00±0.06	1.51±0.13	0.0039
Feed efficiency during pregnancy. g/kJ	2.00±0.33	7.12±0.48	<0.0001
Body mass (at 10-days lactational period), g	19.70±0.42	22.90±0.44	0.0002
Adiposity index (at 10-days lactational period), %	3.4±0.17	4.1±0.20	0.03
Food intake during lactational period, g/day/mouse	3.2±0.20	3.4±0.14	ns
Energy intake during lactational period, kJ/day/mouse	52.80±1.10	69.30±2.92	0.0002
OGTT before experimental diets, AUC, a.u.	13,205.0±364.0	14,273.0±895.0	ns
OGTT after 8-weeks of experimental diets, AUC, a.u.	11,843.0±536.0	16,890.0±762.0	0.0006
Fasting glucose, mmol/L	4.7±0.2	5.7±0.2	0.0077
Cortiscosterone, ng/mL	2.89±0.19	5.25±0.20	<0.0001
Insulin, pg/mL	203.2±4.9	276.7±24.4	0.018

*P*-values of the differences between groups are indicated (unpaired *t*-test). Legend: OGTT = oral glucose tolerance test; AUC = area under the curve; a.u. = arbitrary units; ns = not significant.

**Table 2:** Data from offspring of the mothers fed standard chow (SC) or high-fat diet (HF).

Data	Offspring		
	SC	HF	*P*-value
**Day 0 (birth day)**			
Male∶female ratio	1∶1	1∶1	ns
Litter offspring number	7±1	6±1	ns
Mean offspring mass, g	1.30±0.03	1.44±0.08	ns
Number of dead pups per dam group, %	3/126	16/102	0.0013
**Three-mo-old**			
OGTT, AUC, a.u.	15,320.0±689.0	19,554.0±493.0	0.0011
Insulin, pg/mL	102.2±11.04	204.8±9.93	0.0001

Data are expressed as mean and SEM. *P*-values of the differences between groups are indicated (unpaired *t*-test or Fisher exact test). Legend: OGTT = oral glucose tolerance test; AUC = area under the curve; a.u. = arbitrary units; ns = not significant.

In regard to the BM at 10 days into the lactational period, HF dams were heavier than SC dams (+16%, 22.90±0.44 g *vs.* 19.70±0.42 g, *P* = 0.0002), and they exhibited a greater energy intake (+31%, 69.3±2.9 kJ *vs.* 52.8±1.1 kJ, *P*<0.05). However, there was no difference in food intake (in grams) detected between the HF and SC dams. Moreover, the HF dams showed a higher adiposity index than the SC dams (+21%, 4.1±0.20% *vs.* 3.4±0.17%, *P*<0.05) (Table 1).

#### OGTT

OGTTs performed before diet administration showed no significant differences between the SC and HF dams. However, after eight weeks on the respective diets, the HF dams exhibited higher levels of fasting glucose than the SC dams (+21%, 5.7±0.2 mmol/L vs.. 4.7±0.2 mmol/L, *P* = 0.0017). Additionally, the HF dams presented a 43% higher OGTT AUC (16, 890.0±762.0 a.u.) than the SC dams (11, 843.0±536.0 a.u.) (*P* = 0.0006) (Table 1).

#### Blood pressure, serum corticosterone, and insulin

**Figure 4:**
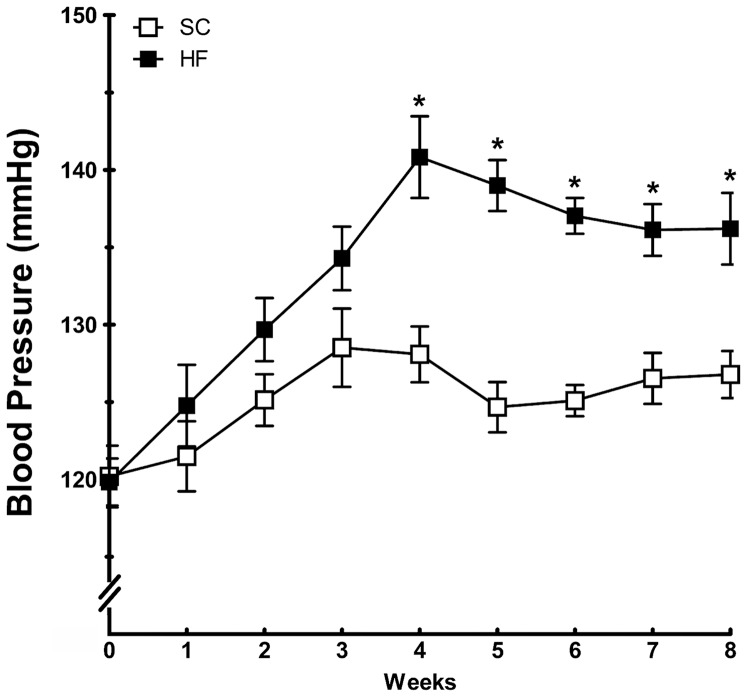
Systolic blood pressure evolution in mothers before pregnancy (mean and standard error of the mean). Significant differences (*P*<0.05) are indicated with an asterisk (n = 10 animals each group, unpaired *t*-test). Abbreviations: SC, standard chow group; HF, high fat diet group.

Figure 4 shows the BP changes in the period before mating. BP was significantly elevated in dams fed an HF diet compared with dams fed SC. Differences in BP were observed as early as the fourth week after diet adjustment and continued until mating (*P<*0.0001).

The serum corticosterone concentration in SC dams was 2.89±0.19 ng/ml, whereas that in HF dams was 82% higher at 5.25±0.20 ng/ml (*P<*0.0001, unpaired *t*-test) (Table 1).

The HF dams showed higher insulin concentrations than the SC dams (+36%, 276.7±24.4 pg/ml vs. 203.2±4.9 pg/ml, *P*<0.018) (Table 1).

### Offspring

The neonatal mortality was higher in the HF offspring compared to the SC offspring (*P* = 0.0013), though this did not compromise the final number of HF offspring. The litters were standardized to seven pups per dam, and only one male from each litter was randomly assigned to each of the experimental groups (Table 2).

#### Body mass and blood glucose

**Figure 5:**
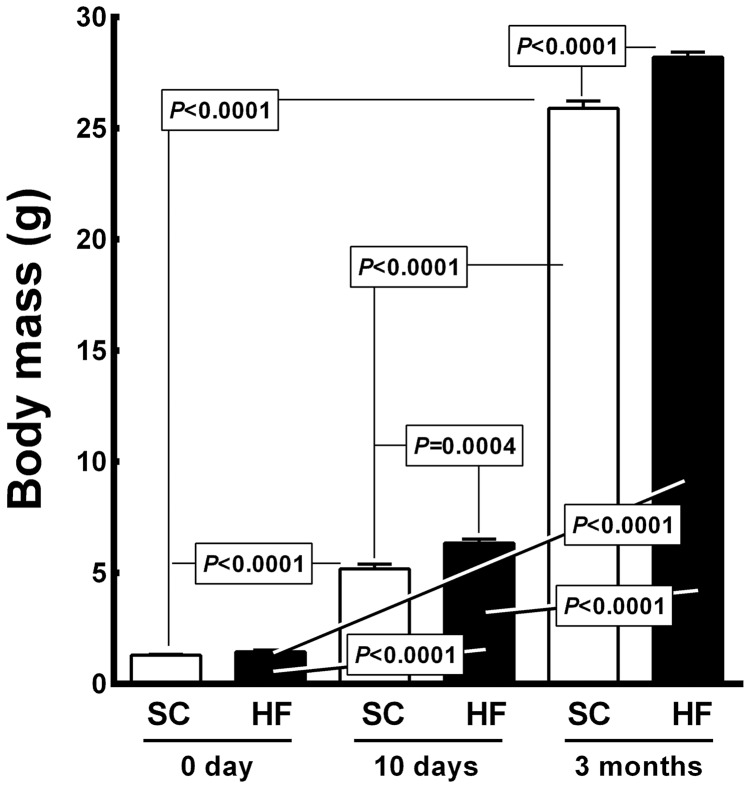
Offspring body mass (mean and standard error of the mean). Significant differences are indicated (one-way ANOVA and post-hoc Holm-Sydak test). Abbreviations: SC, standard chow group; HF, high fat diet group.

Figure 5 shows the BM data for the offspring. At birth, the BM did not differ between the SC pups and HF pups (6.34±0.18 g *vs.* 5.18±0.20 g). However, at 10 days of age, the HF offspring weighed 22% more than the SC offspring (*P = *0.0004), and both the SC and HF offspring showed a significantly elevated BM compared to their respective BM values at birth (+300% for SC offspring, 5.18±0.20 g *vs.* 1.29±0.037 g, *P<*0.0001; +340% for HF offspring, 6.34±0.18 g *vs.* 1.44±0.081 g, *P<*0.0001). At 3 months of age, the HF offspring were heavier than the SC offspring (+9%, 28.2±0.23 g *vs.* 25.9±0.32 g, *P*<0.0001). Compared to the day 0 and day 10 groups, the SC offspring at 3 months of age exhibited BM growth of 1, 907% (from 1.29±0.037 g to 25.9±0.32 g, *P*<0.0001) and 400% (from 5.18±0.20 g to 25.9±0.32 g, *P*<0.0001), respectively. The HF offspring at 3 months of age presented BM growth of 1, 858% compared to day 0 (from 1.44±0.081 g to 28.2±0.23 g, *P<*0.0001) and 345% compared to the day 10 offspring (from 6.34±0.18 g to 28.2±0.23 g, *P<*0.0001). The diet accounted for 0.29% of the differences observed in BM and was significant (*P<*0.0001, two-way ANOVA). The age of the pups accounted for 99.54% of the differences detected in BM and was significant (*P<*0.0001, two-way ANOVA). Moreover, there was an interaction between diet and age accounting for 0.15% of the total variance; this interaction was also significant (*P<*0.0001, two-way ANOVA).

**Figure 6:**
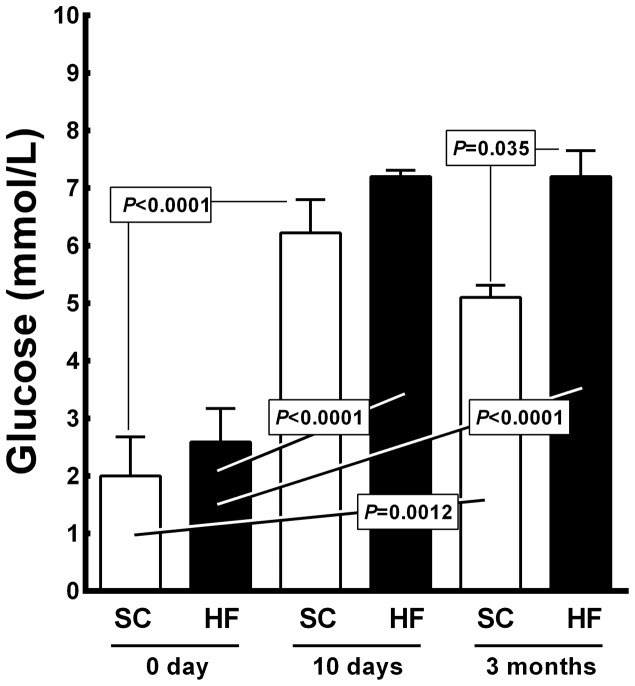
Offspring blood glucose levels (mean and standard error of the mean). Significant differences are indicated (one-way ANOVA and post-hoc Holm-Sydak test). Abbreviations: SC, standard chow group; HF, high fat diet group.

Figure 6 shows the blood glucose levels (BGL) recorded in the offspring. The BGL for 10-day-old SC and HF pups differed from their respective BGL at birth (+211% for SC pups, 6.22±0.26 mmol/L *vs.* 2.00±0.30 mmol/L, *P*<0.0001; and +178% for HF pups, 7.20±0.05 mmol/L *vs.* 2.60±0.26 mmol/L, *P<*0.0001). At 3 months of age, the HF offspring exhibited higher BGL than the SC offspring (+41%, 7.2±0.45 mmol/L *vs.* 5.1±0.21 mmol/L, *P* = 0.035). At 3 months of age, the BGL of both the SC and HF offspring differed from their respective BGL at birth (+155% for SC offspring, from 2.00±0.30 mmol/L to 5.1±0.21 mmol/L, *P* = 0.0012; +178% for HF offspring, from 2.60±0.26 mmol/L to 7.2±0.45 mmol/L, *P*<0.0001). The diet accounted for 8.29% of the BGL differences, which was significant (*P<*0.0001, two-way ANOVA). Pup age accounted for 85.33% of the differences observed in BGL, which was also significant (*P<*0.0001, two-way ANOVA). There was an interaction detected between diet and age, accounting for 2.27% of the total variance, which was a statistically significant difference (*P<*0.05, two-way ANOVA).

The OGTT conducted at 3 mo of age showed significantly higher OGTT results in the HF offspring than in the SC offspring (AUC +28%, from 15, 320.0±689 a.u to 19, 554.0±493 a.u, *P*<0.001). Moreover, even in the animals fed an SC diet from weaning to 3 months of age, the insulin level was 100% higher in the HF offspring than in the SC offspring (from 102.2±11.0 pg/mL to 204.8±9.3 pg/mL, *P*<0.0001) (Table 2).

### Pancreas

**Figure 7:**
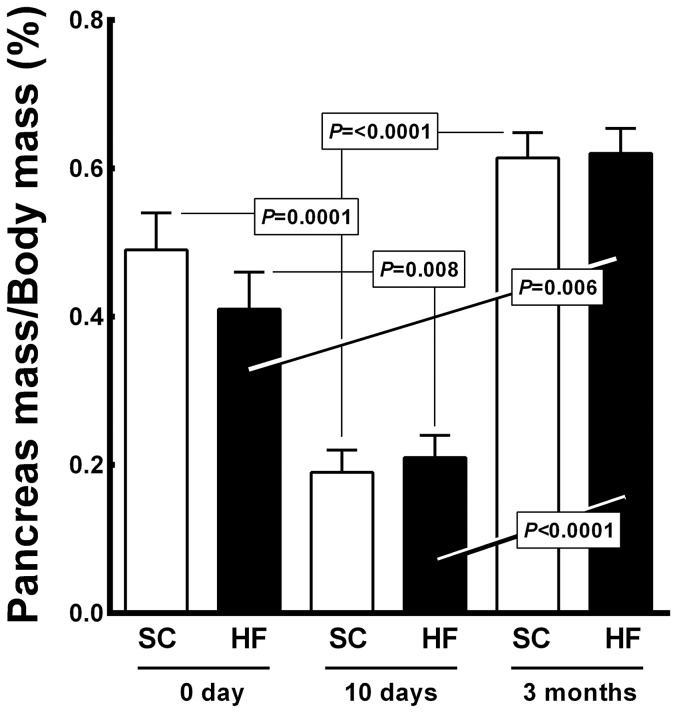
Offspring pancreas mass/body mass ratio (mean and standard error of the mean). Significant differences are indicated (one-way ANOVA and post-hoc Holm-Sydak test). Abbreviations: SC, standard chow group; HF, high fat diet group.

Figure 7 shows pancreas mass/body mass (PM/BM) ratio. Ten-day-old offspring from both groups showed a reduced PM/BM ratio compared with their respective PM/BM ratios at birth (61% decrease for SC pups, 0.19±0.01% *vs.* 0.49±0.02%, *P = *0.0001; 49% decrease for HF pups, 0.21±0.01% *vs.* 0.41±0.02%, *P = *0.008). However, at 3 months of age, the SC offspring exhibited a higher PM/BM compared to the ratio recorded at 10 days of age (+221%, from 0.19±0.03% to 0.61±0.03%., *P*<0.0001). At 3 months of age, the HF pups presented a higher PM/BM than both the HF pups at birth (+51%, from 0.41±0.02% to 0.62±0.03%, *P* = 0.006) and the HF pups at 10 days of age (+195%, from 0.21±0.01% to 0.62±0.03%, P<0.0001). Diet was not a significant factor in the PM/BM ratio (*P>*0.05, two-way ANOVA), but age accounted for 94.27% of the differences observed in the PM/BM ratio and was significant (*P<*0.0001, two-way ANOVA). The interaction between diet and age accounted for 1.57% of the differences observed and was statistically significant (*P<*0.01, two-way ANOVA).

**Figure 8:**
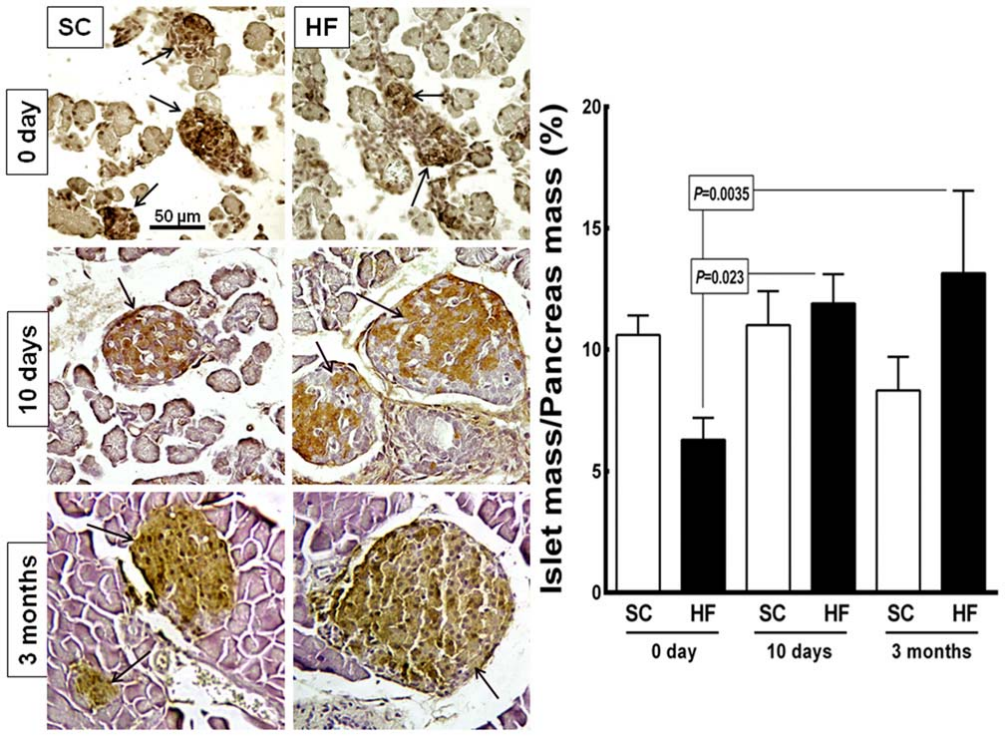
Offspring pancreas. Left side: microscopic images of the pancreas tissue immunostained with an anti-insulin antibody (beta cells, same magnification in all pictures). In the pancreata of mice at birth, we observed small dense islets (arrows) embedded in loose tissue in the both SC and HF groups. On postnatal day 10 and further at 3 months of age, the islets are more organized than at birth. The islets are larger in the HF offspring than in the SC offspring. Right side: bar graph of the islet mass/pancreas mass ratio (mean and standard error of the mean). Significant differences are indicated (one-way ANOVA and post-hoc Holm-Sydak test). Abbreviations: SC, standard chow group; HF, high fat diet group.

Figure 8 shows representative images of the pancreas tissue and islets stained with the anti-insulin antibody (beta cells) as well as the islet mass/pancreas mass (IM/PM) ratio. Strikingly, the mean diameter of the islets in the HF offspring had increased by 155% at 10 days of age (from 40.0±3.9 µm at birth to 101.9±18.4 µm at 10 days of age, *P = *0.0005) and by 195% at 3 mo of age compared with at birth (from 40.0±3.9 µm to 118.20±1.8 µm, *P*<0.0001). An increase in the mean diameter of the islets by 72% was observed in the SC offspring from birth to 3 mo of age (from 52.5±7.9 µm to 90.4±2.3 µm, *P* = 0.04). The differences were not significant when the islet diameter was compared between the SC and HF offspring in all age groups. Diet accounted for 7.05% of the differences observed in the islet diameter (*P = *0.0001, two-way ANOVA). Age accounted for 73% of the differences in islet diameter, which was significant (*P<*0.0001, two-way ANOVA). The interaction between diet and age accounted for 11.8% of the differences detected and was significant (*P<*0.0001, two-way ANOVA).

In the HF offspring, the IM/PM ratio increased by 88% from birth to 10 days of age (from 6.3±0.5% to 11.9±0.8%, *P = *0.023) and by 107% from birth to 3 months of age (from 6.3±0.5% to 13.14±1.52%, *P* = 0.0035). The diet did not have a significant effect on the IM/PM ratio (*P = *0.26, two-way ANOVA). However, age accounted for 26.50% of the differences observed and was significant (*P<*0.0001, two-way ANOVA). Moreover, the interaction between diet and age accounted for 56.35% of the differences in the IM/PM ratio, which was significant (*P<*0.0001, two-way ANOVA).

**Figure 9:**
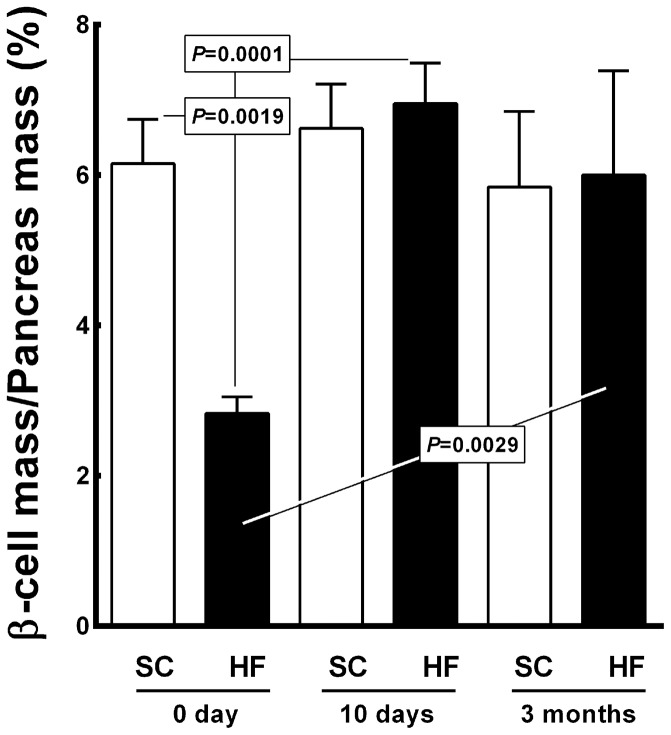
Offspring beta cell mass/pancreas mass ratio (mean and standard error of the mean). Significant differences are indicated (one-way ANOVA and post-hoc Holm-Sydak test). Abbreviations: SC, standard chow group; HF, high fat diet group.

Figure 9 shows the results regarding the beta cell mass/pancreas mass (BCM/PM) ratio. The BCM/PM ratio was 54% lower in the HF offspring than in the SC offspring at birth (2.8±0.1% *vs.* 6.1±0.3%, *P = *0.0019). No differences were observed for the BCM/PM ratio between the HF and SC groups at 10 days of age. In the HF offspring, the BCM/PM ratio increased by 146% from birth to 10 days of age (from 2.8±0.1% to 6.9±0.5%, *P = *0.0001) and by 112% from birth to 3 months of age (from 2.8±0.1% to 6.00±0.62%, *P* = 0.0029). Diet accounted for 17.46% of the differences observed in the BCM/PM ratio and was significant (*P = *0.0006, two-way ANOVA). Age accounted for 41.14% of the total variance and was also significant (*P<*0.0001, two-way ANOVA). In addition, there was interaction between diet and age, which accounted for 26.01% of the differences detected in the BCM/PM ratio, which was considered significant (*P<*0.0001, two-way ANOVA).

**Figure 10:**
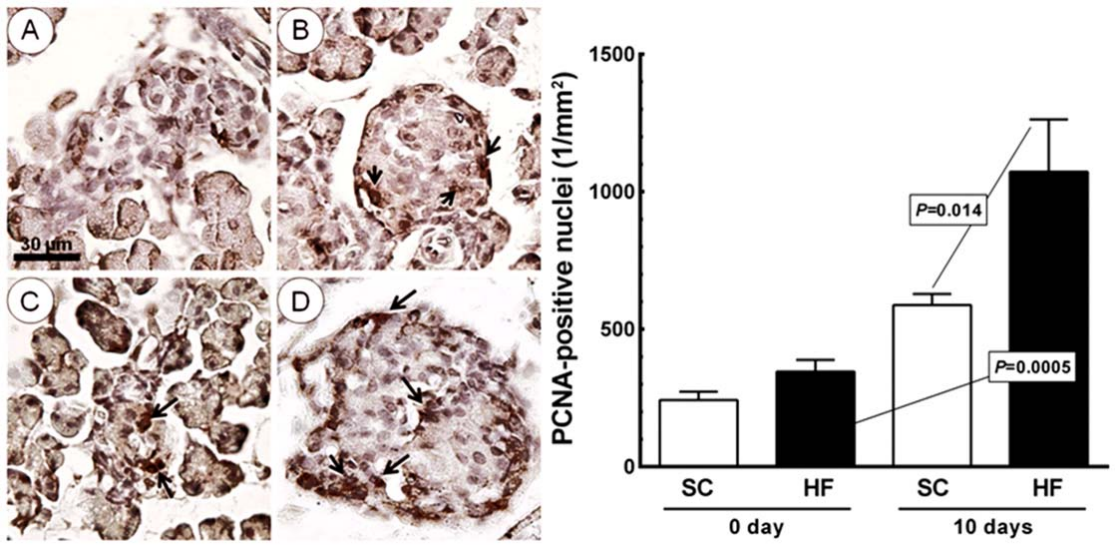
Offspring islets on the day of birth (A, SC group; C, HF group) and at 10 days of age (B, SC group; D, HF group) immunostained with anti-proliferating cell nuclear antigen (PCNA) (arrows, same magnification for all pictures). Right side: bar graph of PCNA density per area in the islet (mean and standard error of the mean). Significant differences are indicated (one-way ANOVA and post-hoc Holm-Sydak test). Abbreviations: SC, standard chow group; HF, high fat diet group.

Figure 10 shows the islet PCNA density per area, which did not differ between the SC and HF offspring at day 0 or in SC offspring from day 0 to day 10. In the HF offspring, the PCNA density per area increased by 210% from day 0 to day 10 (from 346.0±95.4/mm^2^ to 1, 072.7±425.4/mm^2^, *P* = 0.0005). The PCNA nuclear density per area was 82% greater at day 10 in the HF offspring than in the SC offspring (1, 072.7±425.4/mm^2^ vs. 588.2±89.7/mm^2^, *P* = 0.014).

**Figure 11:**
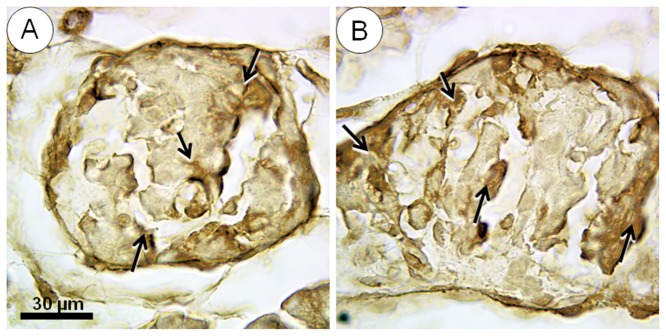
Offspring islet Pdx1 (Pancreatic and duodenal homeobox 1) immunostainning (arrows) on the day of birth. (A) Standard chow group, (B) high fat diet group. No significant differences were observed between these groups.

Figure 11 presents the islet Pdx1 immunostaining results, which did not show significant differences between the groups of offspring early in life.

**Figure 12:**
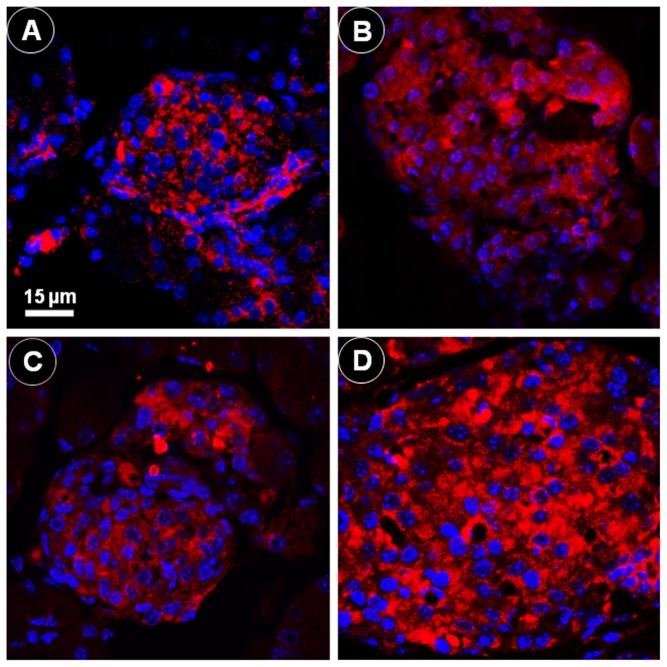
Offspring islets. Positive immunofluorescence reaction for insulin (red color) and DAPI nucleic acid staining (blue color), observed via confocal laser scanning microscopy: (A) standard chow (SC) group, day 0; (B) SC group, day 10; (C) high fat diet (HF) group, day 0; (D) HF group, day 10 (same magnification in all pictures). The images were used to measure the inter-cell nuclear distance as a measure of the beta cell size characterizing cell hypertrophy. The distance increased significantly from day 0 to day 10 in the HF group.

Figure 12 shows the positive immunofluorescence reaction for insulin and DAPI nucleic acid staining. As noted in the Materials and Methods section, we used the inter-cell nuclear distance (ICND) as a measure of the beta cell size indicating cell hypertrophy. In the HF offspring, the ICND increased up 24.1% between the day of birth and the 10 days of age (from 5.23±0.734 µm to 6.49±0.767 µm, *P* = 0.0008). In the SC offspring, the ICND did not show a significant difference between the day of birth and the 10 days of age (from 4.28±0.0489 µm to 4.34±0.422 µm, *P*>0.05).

## Discussion

Mothers fed an HF diet for several weeks before gestation, during gestation and during lactation exhibited an increase in BM, which has been shown in previous studies [27], [28]. The feed efficiency followed a pattern similar to the BM gain, confirming the efficacy of a high-energy diet in increasing adiposity. The consumption of an HF diet prior to and during pregnancy significantly increased the maternal BM relative to mice fed SC prior to and during pregnancy and at day 10 of the lactational period. Although the HF diet influenced the maternal BM, this did not occur considering the total mass of pups in the litter as well as the number of pups in the litter. In addition, pre-gestational exposure of mothers to the HF diet had the consequence of altering the pancreas structure in neonatal mice, which was more evident in adult mice. Moreover, the HF diet-fed mothers showed an increase in serum corticosterone levels and BP when compared with the SC mothers. The OGTT results obtained before and during pregnancy also indicated the existence of glucose intolerance in HF mothers during pregnancy. Therefore, at day10 in the lactational period, the HF dams showed a higher adiposity index compared to the SC dams as well as higher levels of insulin. These data show that mothers fed an HF diet experience significant BM gains, BP elevation and glucose intolerance, which could affect the pancreas and insulin resistance in their offspring.

Previous animal models of neonatal overnutrition have demonstrated a link between excessive BM gains early in life and later metabolic complications [29]. Chronic maternal consumption of an HF diet is associated with insulin resistance in the progeny due to various causes, including the fact that HF diet consumption impairs glucose uptake in the skeletal muscle [30], [31], alters insulin signaling [32], leads to beta cell failure [33], [34] and promotes obesity [35]. An HF diet is strongly associated with insulin resistance and type 2 diabetes [36]. In fact, an increase in the fat mass in an obese state induces the development of glucose intolerance, insulin resistance and hypertension [27], [37]. Excessive adipose tissue also activates the renin-angiotensin aldosterone system, contributing to BP elevation [38]. It is remarkable that rodents fed a cafeteria-type diet for one month prior to breeding and during the gestational and lactational periods do not show changes in blood glucose levels but still become glucose intolerant during pregnancy based on a glucose tolerance test [39].

Our results showed elevated serum corticosterone levels in HF dams. The consumption of an HF diet during development affects the complex interactions between nutrient uptake, metabolism and neuroendocrine systems responsible for homeostasis during critical periods of development in offspring once they reach adulthood [40]. In adult rats, elevation of the plasma concentration of free fatty acids causes marked increases in circulating levels of adrenocorticotropic hormone and corticosterone in response to HF stress [41]. This finding is consistent with our observation that mice fed an HF diet exhibit higher corticosterone levels than mice fed an SC diet.

At birth, the BM was not significantly different between the SC and HF offspring. Although studies have shown that there is no difference in the BM at birth in the offspring of mothers fed an HF diet compared with SC-fed mothers [6], [42], [43], maternal hyperglycemia may induce hyperinsulinemia in the offspring and has functionally teratogenic significance for a predisposition to obesity and diabetes [44]. However, at both 10 days and 3 mo of age, the HF offspring weighed more than the matched SC offspring, indicating the effect of a maternal HF diet during early postnatal life and adulthood influencing the development of obesity in the offspring [5], [7], [45]. Importantly, we observed a significant interaction between a maternal HF diet and offspring BM, suggesting that a maternal HF diet predisposes the offspring to become overweight. An increase in body fat, particularly in white adipose tissue, is an early indicator of obesity, which precedes the development of fatty liver disease and insulin resistance [46], [47].

In the present study, mothers fed an HF diet birthed offspring in which significant structural remodeling of the pancreas occurred, effectively impairing glucose metabolism. Beta cell proliferation occurs at a high rate near the end of embryogenesis, which leads to a massive increase in beta cell mass. The increases in beta cell mass slow down considerably in adult animals, although variations in insulin demands due to physiological and pathological states such as pregnancy and obesity can lead to adaptive changes in the beta cells, such as hyperplasia, hypertrophy, and increased insulin synthesis and secretion [48]. We showed that newborn HF offspring exhibit decreases in beta cell mass and insulin secretion, but without alterations in Pdx1 or PCNA immunostaining patterns. Islet transduction using dominant-negative Pdx1 impairs mitochondrial metabolism and glucose-stimulated insulin secretion [49], and Pdx1 has anti-apoptotic and proliferative activities that help facilitate the maintenance of beta cell mass [50]. However, mechanisms other than proliferation underlie the rapid beta cell growth response following a mild glucose infusion in normal rats, which involve an Akt-regulated enhanced beta cell survival potential and neogenesis from epithelial precursors [51]. Excess fetal glucocorticoids and glucocorticoid receptor signaling are key factors in progenitor cells that program beta cell mass and dysfunction [52].

Beta cell mass is increased by beta cell neogenesis, beta cell proliferation and beta cell hypertrophy, whereas beta cell mass is reduced by beta cell death, primarily through apoptosis and hypotrophy [53]. Decreases in any of the pathways involved in beta cell formation or increases in the rate of beta cell death result in a decreased beta cell mass, which would, in turn, cause reduced insulin output [54]. Elevated glucose and free fatty acid concentrations have deleterious effects on beta cell development and functions [36], [55], resulting in beta cell apoptosis [56], [57]. In *in vivo* rodent models and *in vitro* cell-based models using beta cells, elevated glucose levels can cause increased beta cell apoptosis, resulting in insulin deficiency [58]. Fatty acids have been shown to induce lipotoxicity and beta cell apoptosis through endoplasmic reticulum stress [58], [59]. In addition, the effect of dietary fat in the pancreas may enhance islet amyloid formation and play a role in the production of islet lesions typical of type 2 diabetes [60]. Furthermore, our results showed that there was an increase in corticosterone levels and elevated BP in mothers fed an HF diet. Glucocorticoids appear to play a key role in decreasing the fetal pancreatic beta cell mass by acting on specific receptors located within beta cells, thus resulting in a reduced beta cell mass during early fetal development [60]–[62]. We demonstrated that the beta cells underwent hypertrophy from day 0 to 10 days of age, as the inter-nuclear distance increased within the pancreatic islets marked with an anti-insulin antibody. This finding is strongly suggestive of growth of the entire cell and was significantly different in the offspring from the mothers fed the HD diet.

We observed no significant differences in pancreatic morphology between the SC and HF offspring at 10 days of age and at 3 mo of age. However, the HF offspring were heavier at 10 days of age and at 3 mo of age, and they presented high blood glucose levels compared with the SC offspring at 3 mo of age. Although our results did not show an increase in blood glucose levels in the HF offspring at 10 days of age, a previous study demonstrated a hyperglycemic state in HF offspring at weanling [61]. Endocrine pancreatic plasticity can be defined as the ability of the pancreas to adapt the beta cell mass to variations in the insulin demand to achieve optimal glucose control [62]. A loss of beta cell mass early in organismal development might lead to dysfunction of the remaining beta cells due to over-stimulation or the toxic effects of chronic hyperglycemia and/or hyperlipidemia [40]. The hyperglycemic state displayed in the HF offspring may represent an adaptive pancreatic response.

Animal models showing a reduction in beta cell mass induced by nutrient stress exhibit compensatory adaptation in the remaining beta cells. Hypertrophy and increased insulin secretion in response to glucose and free fatty acid levels may also occur in the remaining beta cells. The adaptive pancreatic response could be insufficient or temporary because of the incomplete differentiation of newly formed beta cells and/or dysfunction of the residual beta cells due to being chronically exposed to a metabolically altered environment [63]. In the present study, differences in beta cell mass were evident at birth but had normalized by the end of organogenesis (10 days of age).

Our principal findings strongly suggest that exposure to a maternal high fat diet during the pre- and perinatal period does have long-term effects on the life of adult offspring, and the altered pancreatic structure during the early neonatal period might be a significant contributor to insulin resistance. Evidence shows that a child of an obese mother may suffer from exposure to a suboptimal *in utero* environment and that the resultant early life adversities may extend into adulthood [64], [65]. Exposure to a maternal HF diet before gestation, during gestation and during lactation affects the metabolic phenotype of the offspring in early and adult life, leading to changes in pancreas structure and, in turn, to altered metabolic function. Animal studies have provided some evidence to support this mode of transgenerational non-Mendelian inheritance [24]. Studies in animals are now addressing the hypothesis that elements of obesity-related metabolic dysfunction in the mother may lead to an altered DNA methylation or acetylation status or to an altered histone structure in the offspring. Such epigenetic processes may contribute, for example, to the recently described altered hepatic expression of IGF-2 and key microRNAs in adult offspring of mice exposed *in utero* and during lactation to a maternal fat rich diet [66].

In the present study, the adult offspring of HF mothers showed hyperinsulinemia compared with SC offspring, supporting an influence of diet-induced maternal obesity before gestation, during gestation and during lactation on pancreatic dysfunction in adulthood. Notably, recent studies in obese pregnant women have shown a similar association between maternal adiposity and insulin resistance and fetal adiposity and insulin resistance, suggesting that metabolism may already be compromised at birth in infants of obese mothers [67], [68].

In conclusion, our findings demonstrate that exposure to a maternal HF diet during the preconceptional period and throughout the gestational and lactational periods in mice results in dramatic alterations in the pancreata of the offspring. The observed pancreatic differences include physiological remodeling of the pancreas and beta cell hypertrophy, which effectively normalizes the beta cell mass by the end of organogenesis but could affect pancreatic function later in life. Our results suggest a pivotal role of maternal nutrition in the development of insulin resistance and diabetes in the offspring once they reach adulthood.
